# Optimization of Ultrasonic-Assisted Enzymatic Extraction Conditions for Improving Total Phenolic Content, Antioxidant and Antitumor Activities In Vitro from *Trapa quadrispinosa* Roxb. Residues

**DOI:** 10.3390/molecules22030396

**Published:** 2017-03-06

**Authors:** Feng Li, Yi-Dan Mao, Yi-Fan Wang, Aun Raza, Li-Peng Qiu, Xiu-Quan Xu

**Affiliations:** 1 Department of Cardiothoracic Surgery, Affiliated Hospital of Jiangsu University, Zhenjiang 212001, China; lifengjs@126.com; 2 School of food and biological engineering, Jiangsu University, Zhenjiang 212013, China; 3 School of Pharmacy, Jiangsu University, Zhenjiang 212013, China; danyimao123@163.com (Y.-D.M.); 15751002880@163.com (Y.-F.W.); aunraza1990@hotmail.com (A.R.); 4 Institute of life sciences, Jiangsu University, Zhenjiang 212013, China; tulip_lipeng@163.com

**Keywords:** *Trapa quadrispinosa* Roxb., stems, ultrasonic-assisted enzymatic extraction, antioxidant activity, antitumor activity, response surface methodology

## Abstract

Stems are the important residues of *Trapa quadrispinosa* Roxb., which are abundant in phenolic compounds. Ultrasonic-assisted enzymatic extraction (UAEE) is confirmed as a novel extraction technology with main advantages of enhancing extraction yield and physiological activities of the extracts from various plants. In this study, UAEE was applied to obtain the highest yield of phenolic content, strongest antioxidant, and antitumor activities and to optimize the extraction conditions using response surface methodology (RSM). The extracts from the stems of *T. quadrispinosa* were characterized by determination of their antioxidant activities through 2,2-azinobis(3-ethylbenzthiazoline)-6-sulfonic acid (ABTS), 1,1-Diphenyl-2-picrylhydrazxyl (DPPH) radical scavenging, total antioxidant capacity (TAC), ferric reducing antioxidant capacity (FRAC) methods and of their antitumor activity by MTT method. The selected key independent variables were cellulase concentration (*X*_1_: 1.5%–2.5%), extraction time (*X*_2_: 20–30 min) and extraction temperature (*X*_3_: 40–60 °C). The optimal extraction conditions for total phenolic content (TPC) value of the extracts were determined as 1.74% cellulase concentration, 25.5 min ultrasonic extraction time and 49.0 °C ultrasonic temperature. Under these conditions, the highest TPC value of 53.6 ± 2.2 mg Gallic acid equivalent (GAE)/g dry weight (DW) was obtained, which agreed well with the predicted value (52.596 mg GAE/g·DW. Furthermore, the extracts obtained from UAEE presented highest antioxidant activities through ABTS, DPPH, TAC and FRAC methods were of 1.54 ± 0.09 mmol Trolox equivalent (TE)/g·DW; 1.45 ± 0.07 mmol·TE/g·DW; 45.2 ± 2.2 mg·GAE/g·DW; 50.4 ± 2.6 μmol FeSO_4_ equivalent/g·DW and lowest IC_50_ values of 160.4 ± 11.6 μg/mL, 126.1 ± 10.8 μg/mL, and 178.3 ± 13.1 μg/mL against Hela, HepG-2 and U251 tumor cells, respectively. The results indicated that the UAEE was an efficient alternative to improve extraction yield and enhance the antioxidant and antitumor activities of the extracts. The phenolic extracts from the stems of *T. quadrispinosa* had significant antioxidant and antitumor activities, which could be used as a source of potential antioxidant and antitumor agents.

## 1. Introduction

Phenolic compounds, one of the most important secondary metabolites, are greatly distributed in all parts of higher plants [1]. More recently, phenolic compounds have attracted extensive attention due to their numerous biological activities, such as antioxidant, antibacterial, antitumor, anti-diabetic and anti-inflammatory activities [2,3,4,5,6]. In recent years, natural antioxidant phenolic compounds obtained from edible byproducts or residual sources have also become interesting because they could be recycled and developed into new medicinal resources [7,8,9,10].

Extraction of these bioactive phenolic compounds is the first step for their utilization and further research. Traditionally, heating, boiling or refluxing were the most used methods for obtaining these phenolic compounds from plant materials. However, the main drawback of these methods are the loss of phenolic compounds and their bioactivities due to oxidation, ionization and hydrolysis caused by long extraction time and high extraction temperature during extraction process [11,12]. In this context, other innovative extraction techniques, including ultrasonic assisted extraction (UAE), microwave assisted extraction (MAE), enzymatic assisted extraction (EAE) and pressurized assisted extraction (PAE), have been developed [13,14,15,16]. Among them, EAE is considered as a moderate, efficient, and environment-friendly extraction method, and has been proven to be effective in improving the yield of target compounds [15]. However, EAE is usually associated with longer extraction time, which could increase the processing cost [17]. As an alternative, UAE has proven to be a rapid, efficient, simple, and inexpensive method that could give higher reproducibility, greater extraction yields, higher purity of the final product, and little effect on the bioactivities of the products [18,19]. Thus, EAE coupled with ultrasound irradiation may be an effective method for target compounds extraction and many positive results for ultrasonic-assisted enzymatic extraction of target compounds have been reported [20,21,22].

In UAEE, the efficiency of extraction process is influenced by many parameters such as kinds and concentrations of enzymes, solvent composition, solvent to solid ratio, extraction temperature and extraction time. Thus, optimization of these parameters is very important for improving the extraction yields. Response surface methodology (RSM), an effective statistical technique, can investigate and optimize complex processes when the independent variables have combined effect on the response values [18]. The main advantage of RSM is to significantly reduce the number of experimental trials needed for evaluating multiple parameters and their interactions and generating a mathematical model to find the optimal values [23,24].

*Trapa quadrispinosa* also named Sijiaoling, belonging to Trapaceae, are regarded as a popular vegetable for its wonderful flavor and medical functions [25]. The pericarps and stems of *T. quadrispinosa* are commonly discarded as residues. Recent studies have reported that the pericarps exhibit remarkable antioxidant and anticancer activities on behalf of its excellent content of phenolic compounds [26,27]. However, the compositions and biological activities of the stem of *T. quadrispinosa* have rarely been examined.

In our previous study, ultrasonic-assisted extraction has proven to enhance the extraction yield and antioxidant activities of polysaccharides from *T. quadrispinosa* stems [28]. In our further study, phenolic compounds were detected in the stem of *T. quadrispinosa*. However, to the best of our knowledge, there have been no previous reports on the combined use of enzymes and ultrasonic-assisted extraction of phenolic compounds from the stem of *T. quadrispinosa*. Therefore, the aim of this current study was to investigate the feasibility of ultrasonic-assisted enzymatic extraction (UAEE) for the phenolic compounds from the stems of *T. quadrispinosa* and to evaluate their antioxidant and antitumor activities in vitro. Box-Behnken design (BBD) combined with response surface methodology (RSM) was employed to evaluate the effects of different operating parameters on extraction procedure and to optimize the processing conditions.

## 2. Results and Discussion

### 2.1. Single Factor Experiment

In this part, four key parameters including concentration of cellulase, ultrasonic temperature, ultrasonic time and liquid to solid ratio were picked out respectively for investigation.

#### 2.1.1. Effect of Cellulase Concentration on Extraction of TPC

**Figure 1:**
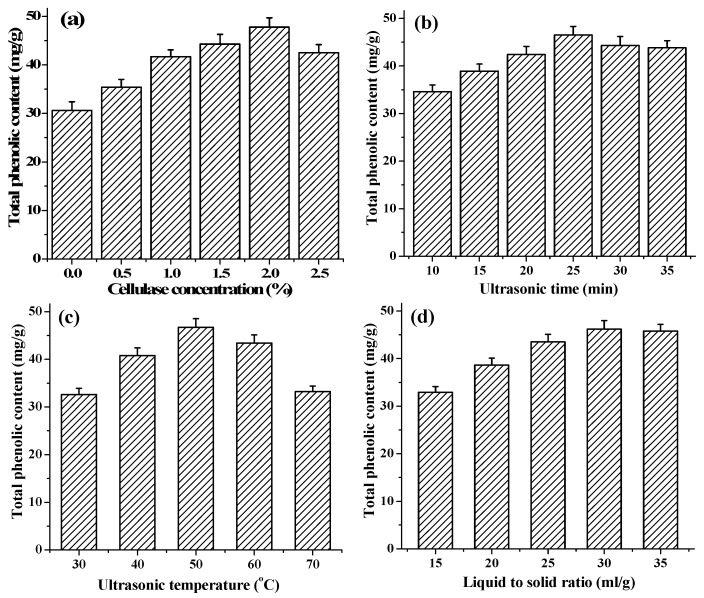
Effects of different extraction parameters on the yield of phenolic extracts: (**a**) cellulase concentration (%); (**b**) extraction time (min); (**c**) extraction temperature (°C); and (**d**) liquid to solid ratio (mL/g).

With UAEE, the enzyme degradation and destruction of the cell-wall matrix have been considered as a primary step to enhance the release of phenolic compounds, keep their stability and antioxidant activities [29]. In this study, with other extraction conditions fixed at ultrasonic time 20 min, ultrasonic temperature 50 °C and liquid to solid ratio 30 mL/g, the effect of cellulase concentrations at different levels (from 0% to 2.5%) on extraction of TPC was studied. As shown in Figure 1a, there was a notable increasing in TPC with increasing cellulase concentrations from 0% to 2.0%, beyond which the TPC value began to decrease. Similar findings were reported for flavonoid extraction from *Illicium verum* residues [30]. This result indicated that 2.0% cellulase could completely hydrolyze the cell walls of *T. quadrispinosa* stems to release the intracellular phenolic compounds. However, the viscous cellulase solution with high concentrations was not avail to the hydrolysis reaction process [30]. Therefore, 2.0% cellulase was considered the optimal enzyme concentration in further experiments.

#### 2.1.2. Effect of Ultrasonic Time on Extraction of TPC

Extraction time is an important parameter that can influence the extraction efficiency. Numerous studies have demonstrated that a long extraction time presented a favorable effect on the production of phenolic compounds [31,32]. However, excessive lengthening of extraction time may induce the change of phenolic compounds molecule structure and bioactivities because of hydrolysis or oxidization [33]. To investigate the effect of ultrasonic time on extraction of TPC, different ultrasonic times, varying from 10 to 35 min, were investigated while other conditions were set as follows: cellulase concentrations 2.0%, ultrasonic temperature 50 °C and liquid to solid ratio 30 mL/g. As shown in Figure 1b, the extraction yield of TPC distinctly increased as the extraction time ascending from 10 to 25 min, the maximum yield of 46.5 ± 1.7 mg·GAE/g was obtained when the extraction time reached 25 min, and, after this point, it began to decrease slightly with a further extension of extraction time. This phenomenon might be due to the degradation of the extracted compounds with excessively lengthening of extraction time [34]. Therefore, extraction time of 25 min was adopted in the present work.

#### 2.1.3. Effect of Ultrasonic Temperature on Extraction of TPC

Extraction temperature is another main important parameter affecting the extraction process. To investigate the effect of temperature on extraction of TPC, various temperatures within 30–70 °C were evaluated in the present study, while keeping the cellulase concentrations 2.0%, ultrasonic time 20 min, and liquid to solid ratio 30 mL/g. As shown in Figure 1c, the TPC value increased positively from 30 °C to 50 °C, and the maximum yield of 46.7 ± 1.8 mg·GAE/g was observed when extraction temperature was at 50 °C. However, the TPC value declined gradually when further increasing extraction temperature above 50 °C. This result was in line with other reports from in extracting resveratrol from *Polygonum cuspidatum* [35]. The decreasing the viscosity coefficient, increasing the diffusion coefficient and enhancing the solubility of phenolics and the better enzyme activities at higher temperatures caused the increase of the phenolic compounds releasing from the stems particles into extraction solution [36,37]. Furthermore, high temperature could decrease number of cavitation bubbles and weaken the impact of cavity collapse on homogenized samples [38]. Meanwhile, high temperatures could also cause thermo degradation or oxidization of phenolic compounds, and denaturalization of cellulase [37,39]. Thus, the optimal extraction temperature was adopted at 50 °C in the present work fixed as the central point for the RSM.

#### 2.1.4. Effect of Liquid to Solid Ratio on Extraction of TPC

Different ratio of liquid to solid is another parameter that could alter the extraction yield of phenolic compounds in UAEE. In this present study, the effect of ratio of liquid to solid in the range of 15 to 35 mL/g on the extraction of TPC was investigated when other parameters were fixed as follows: extraction temperature 50 °C, extraction time 20 min, and cellulase concentration 2.0%, respectively. As shown in Figure 1d, the TPC increased with increasing of ratio of liquid to solid and reached the peak value of 46.2 ± 1.9 mg·GAE/g at liquid to solid ratio of 30 mL/g, and then no longer changed along with the extraction process. The effect of liquid to solid ratio on the yield was not significant (*p* > 0.05). Therefore, this factor was fixed at 30 mL/g and ignored in further experiment.

### 2.2. Response Surface Methodology

#### 2.2.1. The Results in the BBD Experiments

The experimental and predicted values of TPC are listed in Table 1. Among the 15 experiments including three replicates, the TPC values ranged from 44.6 mg·GAE/g·DW to 52.7 mg·GAE/g·DW, and experiment 13 (cellulase concentration 2.0%, ultrasonic time 25 min and ultrasonic temperature 50 °C) has the greatest TPC (52.7 mg·GAE/g·DW) and experiment 8 (cellulase concentration 2.5%, ultrasonic time 25 min and ultrasonic temperature 60 °C) has the smallest (44.6 mg·GAE/g·DW).

#### 2.2.2. Fitting the Model

Based on the experimental data, the obtained model, which shows the relationship between the TPC value of the extracts and the extraction parameters, is presented in the following equations:

*Y*_TPC_ = 52.33 − 0.82*X*_1_ + 0.33*X*_2_ − 0.90*X*_3_ + 0.10*X*_1_*X*_2_ − 0.55*X*_1_*X*_3_ − 0.30*X*_2_*X*_3_ − 0.74*X*_1_^2^ − 1.591*X*_2_^2^ − 4.54*X*_3_^2^ (1)

The statistical significance of the regression model was checked by F-test and *p*-value, and the analysis of variance (ANOVA) for the response surface quadratic model, as shown in Table 2. According to the results, it was observed that quadratic polynomial models were highly significant with the low *p*-values of <0.0001 and the high F values of 74.90 which suggested that the fitness of this model was very high. The high value of determination coefficient (*R*^2^) and adjusted determination coefficient (0.9926 and 0.9794, respectively), very close to 1.0, indicated that there was a good correlation between the experimental and predicted values and only 2.06% of the total variations could not be explained by this model. A relatively low value of C.V. (the coefficient of variation) indicated a better reliability of the experiments values [40]. In this study, C.V. value of 0.78% suggested that the model was accurate and reproducible. Significance of the model was also determined by lack-of-fit test. As shown in Table 2, the *F*-value of 0.80 and *p*-value of 0.5986 suggested that it was not significant and a 59.86% chance could occur due to noise.

The *p*-value was used as a tool to examine the significance of each coefficient, and the smaller of the *p*-value was, the more significant the corresponding coefficient was [41]. It can be seen very clearly in Table 2 that the TPC value was significantly influenced by two linear coefficients (*X*_1_ and *X*_3_), three quadratic coefficients (*X*_1_^2^, *X*_2_^2^, and *X*_3_^2^) and one cross product coefficient (*X*_1_*X*_3_), with small *p*-values less than 0.05. However, other term coefficients did not significantly influence the TPC value with higher *p*-values (*p* values > 0.05).

#### 2.2.3. Analysis of Response Surfaces and Contours

Response surface plots (3D) and contour plots (2D) were represented to evaluate the effects of the independent variables and their interactions on the extraction of TPC. The shapes of the contour plots (circular or elliptical) indicate whether the mutual interactions between the variables were significant. A circular contour plot indicated that the interactions between the corresponding variables were negligible, and an elliptical contour plot indicated that the interactions between the corresponding variables were significant [42].

**Figure 2:**
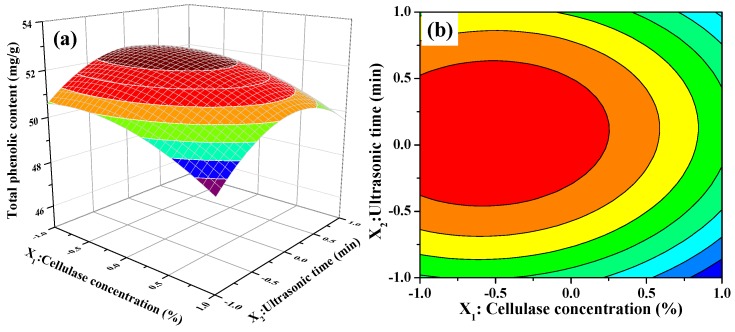

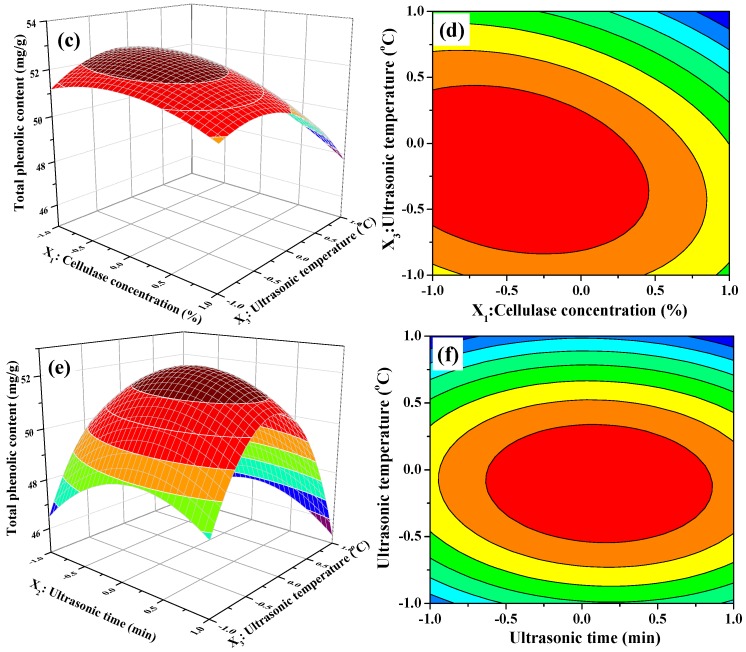
Contour (**a**,**c**,**e**); and response surface (**b**,**d**,**f**) plots for interactions between three independent extraction parameters on the extraction yields of phenolic extracts (*X*_1_: Cellulase Concentration; *X*_2_: Ultrasonic Time; and *X*_3_: Ultrasonic Temperature).

The 3D response surface and 2D contour plots about the relationship between extraction parameters and the value of TPC are presented in Figure 2. Figure 2a,b illustrates the effect of cellulase concentration and ultrasonic time on the TPC value when ultrasonic temperature was fixed at 50 °C (0 level). The extraction of TPC increased rapidly with increasing cellulase concentration from 1.5% to 1.75%, increased slowly with increasing of ultrasonic time from 20 to 26 min, and then followed by a decline with further increase. In Figure 2b, we also observe that the curved surface of cellulase concentration was steeper than the curved surface of the ultrasonic time. The results indicated that both cellulase concentration and ultrasonic time had quadratic effects on TPC value and the influence of cellulase concentration on the extraction of TPC was greater than that of ultrasonic time. Figure 2c,d displays the interaction between cellulase concentration and ultrasonic temperature on the TPC value when ultrasonic time was fixed at 25 min (0 level). When extraction temperature was kept at a lower level, the TPC value initially increased and then decreased slowly with increasing cellulase concentration. Nevertheless, the TPC value significantly decreased when extraction temperature was more than 50 °C. Furthermore, the response value of TPC was influenced significantly by the interaction between the cellulase concentration and ultrasonic temperature because of the elliptical contour shape of the 2-D contour plot. Figure 2e,f describes the interaction between ultrasonic temperature and ultrasonic time on the TPC value at a fixed cellulase concentration of 2.0% (0 level). As presented in Figure 2e, the 3D response surface, the TPC value was very relate to the quadratic effects of ultrasonic temperature and ultrasonic time. The TPC value increased with increasing of ultrasonic temperature or ultrasonic time. However, beyond 0 level, the TPC value decreased obviously with increasing ultrasonic temperature or ultrasonic time when the other one was set. It meant that too low or too high level of ultrasonic temperature or ultrasonic time could decrease the extraction yield and the moderate level of these two extraction parameters resulted in high TPC value. On the other hand, the mutual interaction between ultrasonic temperature and ultrasonic time was not significant demonstrated by the 2-D contour plot shown in Figure 2f.

#### 2.2.4. Verification Experiments

Base on the built mathematical models, the optimal experimental conditions of UAEE for extraction of TPC from the stem of *T. quadrispinosa* was obtained as follows: concentration of cellulase, 1.74%; extraction time, 25.45 min; and extraction temperature, 49.3 °C. Under the optimal parameters, the predictive value of extraction yields was 52.596 mg·GAE/g·DW. Because the values of extraction time and extraction temperature were difficult to operate in the actual extraction experiments, the actual optimal parameters were carried out with slight modifications: concentration of cellulase, 1.74%; extraction time, 25.5 min; and extraction temperature, 49.0 °C. The experimental value of 53.6 ± 2.2 mg·GAE/g·DW agreed well with the predicted values, which confirmed that the response model was adequate to reflect the expected optimization.

### 2.3. Analysis of Microscopic Changes

**Figure 3:**
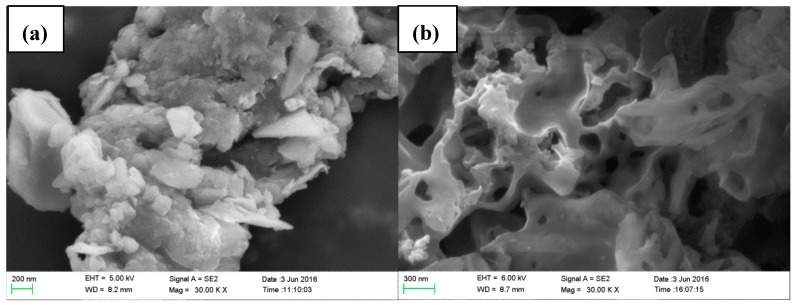
Scanning electron microscopic images of residues in the extraction of *T. quadrispinosa* stems: (**a**) untreated; and (**b**) treated by UAEE.

In order to study the influence of UAEE on the physical changes of the extracted tissue surface and structural features, SEM analysis was applied for observing the microscopic changes. As shown in Figure 3a, it was obvious that there was a complete parenchyma and no destroys on cell walls for the untreated samples. However, samples subjected to UAEE had broken and damaged tissues and many large perforations on the external surfaces were observed (see Figure 3b). These changes could be attributed to the combined effects of cavitation effect brought by ultrasonic vibration and enzymolysis effect provided by cellulase [20].

### 2.4. Comparison of UAEE with Other Extraction Methods

#### 2.4.1. Total Phenolic Content

The efficiency of the TPC value by UAEE and other extraction methods were compared, and the extraction conditions and TPC values used are shown in Table 3. As shown in Table 3, the UAEE method had the highest extraction yield of TPC (53.6 ± 2.2 mg·GAE/g·DW), and the HE had a lowest extraction yield of TPC (42.4 ± 1.3 mg·GAE/g·DW). Compared with HE, UAE and EAE, UAEE had lowest extraction temperature and shortest extraction time with highest extraction yield. The order of yield of TPC extracted with different methods was similar to the results of former studies, which demonstrated that UAEE is more efficient than the three other methods, and is a promising extraction method [21,43].

#### 2.4.2. Antioxidant Activity of Phenolic Extracts

**Figure 4:**
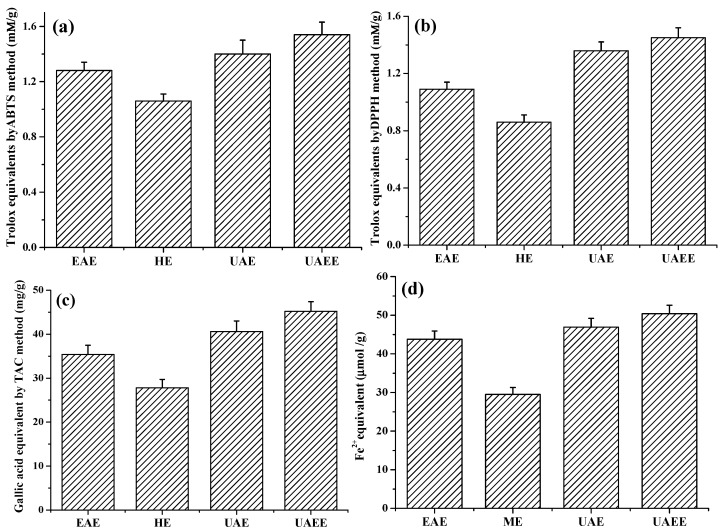
Antioxidant activity of phenolic extracts obtained from different methods: (**a**) ABTS (mmol Trolox equivalent/g dry weight); (**b**) DPPH (mmol Trolox equivalent/g dry weight); (**c**) FRAP (mg Gallic acid equivalent/g dry weight); and (**d**) FRAC (μmol FeSO_4_ equivalent/g dry weight).

Four methods, ABTS, DPPH TAC and FRAC, were chosen to determine the antioxidant capacity of extracts obtained from different extraction methods. The results are shown in Figure 4. As shown in Figure 4, all of the four values exhibited the similar behaviors: UAEE yielded the highest antioxidant activities of 1.54 ± 0.09 mmol·TE/g·DW, 1.45 ± 0.07 mmol·TE/g·DW, 45.2 ± 2.2 mg·GAE/g·DW and 50.4 ± 2.6 μmol FeSO_4_ equivalent/g·DW by ABTS, DPPH, TAC, and FRAP methods respectively. Conversely, HE method yielded the lowest antioxidant activities of 1.28 ± 0.06 mmol·TE/g·DW, 1.09 ± 0.05 mmol·TE/g·DW, 27.8 ± 1.9 mg·GAE/g·DW and 29.5 ± 1.8 μmol FeSO_4_ equivalent/g·DW, respectively.

The above results indicated that the increasing of antioxidant activities of extracts by UAEE process was attributed to the enhancement of the extraction efficiency of phenolic compounds from *T. quadrispinosa* stem residues.

#### 2.4.3. Antitumor Activity of Phenolic Extracts

**Figure 5:**
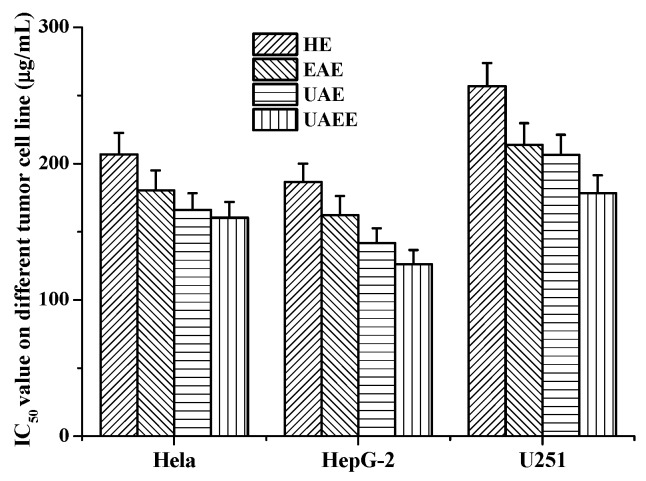
Antitumor activities (IC_50_ μg/mL) of phenolic extracts obtained from different extraction methods on the Hela, HepG-2 and U251 cells.

**Figure 6:**
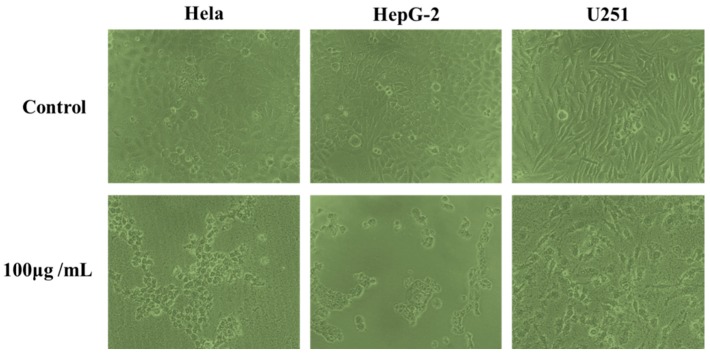
Morphological changes of Hela, HepG-2 and U251 cells treated for 24 h with 100 μg/mL of phenolic extracts of *T. quadrispinosa* stem obtained by UAEE.

The effects of phenolic extracts obtained from different methods on the growth of Hela, HepG-2 and U251cells were investigated using the MTT assay. The results of growth inhibitory effects of different phenolic extracts, with IC_50_ values, are shown in Figure 5, which very clearly indicates all phenolic extracts exhibited high growth inhibitory effects on cell viability of Hela, HepG-2 and U251 cells. Among them, the phenolic extracts obtained from UAEE had greatest inhibitory effect on the growth of cells compared to other methods with the lowest IC_50_ values of 160.4 ± 11.6 μg/mL, 126.1 ± 10.8 μg/mL, 178.3 ± 13.1 μg/mL against Hela, HepG-2 and U251 tumor cells, respectively. However, phenolic extracts obtained from HE had weakest inhibitory effect on the growth of cells with the highest IC_50_ values of 206.8 ± 15.6 μg/mL, 186.5 ± 13.5 μg/mL, and 256.9 ± 16.9 μg/mL against Hela, HepG-2 and U251 tumor cells, respectively. To further confirm the proliferation inhibitory effects on Hela, HepG-2 and U251 cells, the morphology changes induced by phenolic extracts (100 μg/mL) obtained from UAEE were investigated. As illustrated in Figure 6, the morphology of the control untreated cell was regular and intact, whereas cells treated with phenolic extracts exhibited obvious signs of growth inhibition, including decreased cell number, diminished cell volume, and increased cell shrinkage. At the same time, some cells gathered together and dead cells or apoptotic cells appeared. This revealed that phenolic extracts of *T. quadrispinosa* stems could affect cell proliferation and survival which was consistent with the cell viability assay results.

## 3. Material and Methods

### 3.1. Plant Material and Chemical Reagents

The stems of *T. quadrispinosa* were collected from Weishan Lake, Weishan County, Shandong Province, China. The air-dried stems were ground and screened through a 40 mesh sieve for further extraction.

1,1-Diphenyl-2-picrylhydrazxyl (DPPH), 2,2-azinobis(3-ethylbenzthiazoline)-6-sulfonic acid (ABTS), 6-hydroxy-2,5,7,8-tetramethylchroman-2-carboxylic acid (Trolox), 3-(4,5-Dimethyl-2-thiazolyl)-2,5-diphenyl-2*H*-tetrazolium bromide (MTT) and Folin–Ciocalteu’s phenol reagent were obtained from Sigma Chemicals Co. (St. Louis, MO, USA). Dulbecco’s modified eagle medium (DMEM), fetal bovine serum (FBS), Penicillin and Streptomycin were purchased from Gibco Laboratories (Grand Island, NY, USA). Cellulase (EC 3.2.1.1, 400 U/mg), Gallic acid, Sodium carbonate, Iron sulfate heptahydrate, Iron chloride hexahydrate, Ammonium molybdate, Sodium phosphate, Potassium ferricyanide and 1,10-phenanthroline were provided from TianKe Co., Ltd. (Suzhou, China). Ethanol and methanol were obtained from Kelong Chemical Factory (Chengdu, China). All other chemical reagents used in this experiment were of analytical grade and doubly distilled water was used throughout the experiment.

### 3.2. Ultrasonic-Assisted Enzymatic Extraction

The UAEE method has been previously described in the study on the extraction of polyphenols from waste peanut shells [44]. Extractions were carried out in a temperature and time controlled ultrasonic cleaner (KQ-250DB, Kunshan Ultrasonic Co. Ltd., Kunshan, China) with a fixed frequency of 40 kHz and an alterable electric powder range from 100 W to 250 W. The internal tank of the ultrasonic cleaner was 300 mm × 240 mm × 150 mm (L × W × H). In this study, 30% ethanol with pH value of 5.0 adjusted by HCl was chosen as extraction solvent. The stem powders (1.0 g) were placed into 100 mL Erlenmeyer flask. Next, the flask was fixed in the same position in water (the water depth fixed as 100 mm) in the ultrasonic cleaner at 40 kHz, 250 W and extracted according to the experiment design. The designed extraction temperature was adjusted by the temperature controller, and the real temperature of the solution was monitored by a thermometer. After extraction, the mixtures were centrifuged at 4000 rpm for 10 min, and the supernatants were collected for the total phenolic content and further antioxidant and antitumor activity determinations.

### 3.3. Experimental Design

#### 3.3.1. Single-Factor Experiment

To evaluate the effect of each factor on the extraction yield from the *T. quadrispinosa* stems, different cellulase concentration (from 0% to 2.5%), ultrasonic time (from 10 to 35 min), ultrasonic temperature (from 30 to 70 °C) and ratio of liquid to solid (from 15 to 35 mL/g) were investigated as single factors.

#### 3.3.2. Response Surface Methodology Experiment

According the single-factor experimental results, a Box-Behnken Design (BBD) with response surface methodology (RSM) was applied to further determine the optimal UAEE conditions. The levels of the three independent variables were confirmed cellulase concentration (*X*_1_, 1.5%–2.5%), ultrasonic time (*X*_2_, 20–30 min) and ultrasonic temperature (*X*_3_, 40–60 °C) related to the response yield of total phenolic content (mg Gallic acid equivalent/g dried weight). The coded and uncoded (actual) levels of the independent variables were given in Table 4. A quadratic polynomial model performed based on experimental data from CCD was explained by the following quadratic equation:

(2) $$Y=A_{0}+\overset{3}{\underset{i=1}{\sum }}A_{i}X_{i}+\overset{3}{\underset{i=1}{\sum }}A_{ii}X_{i}^{2}+\overset{2}{\underset{i=1}{\sum }}\overset{3}{\underset{j=i+1}{\sum }}A_{ij}X_{i}X_{j}$$

where *Y* is response, *A*_0_ is intercept, *A_i_* is coefficient of variable for linear, *A_ii_* is coefficient of variable for quadratic, and A*_ii_* is coefficient of variable for interaction term. *X_i_* and *X_j_* are independent variables.

The experimental data were analyzed using a statistical package, Design-Expert version 8.0.5b, (Stat-Ease Inc., Minneapolis, MN, USA). The adequacy of the established model and statistical significance of the regression coefficients was evaluated by the lack of fit, coefficient of determination (*R*^2^), and Fisher test value (*F*-value) generated from the ANOVA analysis (*p* < 0.05).

### 3.4. Scanning Electron Microscopy Analysis

In order to investigate the effect of UAEE on the microstructure of samples, the untreated sample as well as residue after extraction of phenolics were collected and air-dried for the scanning electron microscopy (SEM) analysis. The powders were fixed on the aluminum stubs with adhesive tape and covered with gold as a sputter coater. The shape and the surface characters of the powders were examined with a Sigma 500/VP SEM, under high vacuum conditions at a voltage of 10.0 kV.

### 3.5. Comparison with Other Extraction Procedures

#### 3.5.1. Heat Extraction (HE)

*T. quadrispinosa* stem powders (1.0 g) was mixed with 30 mL of 30% ethanol in a 100 mL Erlenmeyer flask. For the extraction, the flask was placed into a water-bath and connected with cooling water, and then allowed to reflux extraction for 120 min at 80 °C.

#### 3.5.2. Ultrasonic-Assisted Extraction (UAE)

*T. quadrispinosa* stem powders (1.0 g) was mixed with 30 mL of 30% ethanol and put into a 100 mL Erlenmeyer flask and was then extracted in an ultrasonic cleaner for 30 min at 50 °C.

#### 3.5.3. Enzyme-Assisted Extraction (EAE)

*T. quadrispinosa* stem powders (1.0 g) was mixed with cellulase solution (2.0%) dispersed in 30 mL of 30% ethanol with pH value of 5.0 in a 100 mL Erlenmeyer flask. For the extraction, the flask was placed into a water-bath, and then allowed to incubation extraction 180 min at 50 °C.

### 3.6. Determination of Total Phenolic Content

The total phenolic content of the extracts was determined by using Folin–Ciocalteu’s reagent described previously [45] with Gallic acid as a standard. Briefly, 200 μL of each suitable diluted extracts was mixed with 0.5 mL of Folin–Ciocalteu’s reagent in 10 mL glass tube. After incubation for 2 min at room temperature, 2 mL of 20% sodium carbonate solution was added and mixed thoroughly. The reaction solution was then brought up to a final volume of 10 mL with distilled water and incubated for 120 min in dark at room temperature. The absorbance of samples was measured at 760 nm. The total phenolic content was expressed as mg of Gallic acid equivalent per gram of dry weight (mg·GAE/g·DW) based on the calibration curve (*Y* = 0.0148*X* − 0.0039, *R*^2^ = 0.9993).

### 3.7. Evaluation of Antioxidant Capacity

ABTS, DPPH TAC and FRAC methods, utilizing the same single electron transfer mechanism, are commonly applied to determine the antioxidant capacity of biological compounds. These four methods were adopted in this study to assess the antioxidant activity of the phenolic extracts from the stems of *T. quadrispinosa.*

#### 3.7.1. ABTS Method

The capacity of the extracts scavenging ABTS^•+^ was carried out according to the procedure described by Sariburun E. [46] with small modification. The radical of ABTS^•+^ was prepared by reacting 7 mM ABTS stock solution with 2.45 mM potassium persulfate in dark conditions at room temperature for 12–16 h. The ABTS^•+^ working solution was obtained by diluting the stock solution with deionized water to an absorbance of 0.7 ± 0.02 at 754 nm. In experiment, 100 μL of suitable diluted extracts was mixed thoroughly with 2.9 mL of diluted ABTS solution, the absorbance of the mixture was measured at 754 nm against blank control after incubation for 6 min at room temperature. Trolox was used as the standard and the results were expressed as mmol Trolox equivalents per gram of dry weight (mmol·TE/g·DW) based on the calibration curve (*Y* = 0.01102*X* − 0.0222, *R*^2^ = 0.9989).

#### 3.7.2. DPPH Method

The DPPH radical scavenging capacity of the extracts was performed using a method described elsewhere [47] with slight modification. A 100 μL of suitable diluted extracts was taken into 10 mL glass tubes and mixed with 2.9 mL of freshly prepared 0.1 mmol DPPH methanol solution. With the mixtures being stood for 30 min at room temperature in dark, the absorbance was measured at 517 nm. The antioxidant capacity was expressed as mmol Trolox equivalents per gram of dry weight (mmol·TE/g·DW) based on the calibration curve (*Y* = 0.00878*X* − 0.0006, *R*^2^ = 0.9998).

#### 3.7.3. TAC Method

The total antioxidant captivity (TAC) of the extracts were evaluated by the phosphomolybdenum method according to the procedure described previously [48] with some modification. A 200 μL of each suitable diluted extracts was mixed with 3.0 mL of complex reagent solution (including 0.6 M sulfuric acid, 28 mM sodium phosphate and 4 mM ammonium molybdate) in 10 mL glass tubes. The reaction solution were incubated at 95 °C for 90 min and then cooled to room temperature. Finally, the absorbance of the solution was measured at 695 nm. The total antioxidant captivity was expressed as mg Gallic acid equivalents per gram of dry weight (mg·GAE/g·DW) based on the calibration curve (*Y* = 0.009*X* + 0.06, *R*^2^ = 0.9992).

#### 3.7.4. FRAC Method

The Ferric reducing antioxidant capacity of extract was determined according to the method of 1,10-phenanthroline [49] with minor modification. Briefly, 200 μL of each suitable diluted extracts was mixed with 0.5 mL of 0.5% 1,10-phenanthroline methanol solution and 1 mL of 0.2% FeCl_3_ methanol solution in 10 mL glass tubes. The reaction solution was then brought up to a final volume of 10 mL with methanol and kept at room temperature in dark for 20 min. Finally, the absorbance of the solution was measured at 510 nm. The result was expressed as μmol of Fe(II) per gram of weight material (μmol·Fe^2+^/g·DW) based on the calibration curve of FeSO_4_ (*Y* = 0.01054*X* − 0.0097, *R*^2^ = 0.9993).

### 3.8. Evaluation of Antitumor Capacity

#### 3.8.1. Cell Culture

The cell lines of Hela, HepG-2 and U251 used in this study were kindly provided by Prof. Jing Gao (School of Pharmacy, Jiangsu University, Su Zhou, China). The cells were cultured in DMEM mediums containing 10% fetal bovine serum, 100 U/mL penicillin, and 100 μg/mL streptomycin under 37 °C in a humidified atmosphere with 5% CO_2_. Cells in log phase were used for experiments.

#### 3.8.2. MTT Cell Proliferation Assay

The cells viability was assessed by MTT colorimetric assay [50]. Briefly, cells were plated into 96-well cell culture plates at an optimum density of 1 × 10^5^ cells/well and incubated for 24 h, and then the cells were treated with serial concentration of the phenolic extracts (50–1000 μg/mL) obtained from different extraction methods for another 24 h. Following treatment, 10 μL of MTT (5 mg/mL) in physiological buffered saline (PBS) was added into each well, and incubated for further 4 h. After adding 150 μL of DMSO to dissolve formed formazan crystals, the absorbance of each well was measured at 570 nm using ELISA reader. The percentage cell viability was calculated using the formulae below: cell viability (%) = OD_570_(Treated sample)/OD_570_(Untreated control) × 100. The IC_50_ values were determined using Graph Pad Prism software (Graph Pad software, 5.0.1, San Diego, CA, USA).

#### 3.8.3. Morphological Evaluation

The cells were incubated in 6-well cell culture plates at a cell density of 2 × 10^5^ cells per well for 24 h. After treatments with 100 μg/mL of the phenolic extracts obtained from UAEE for the subsequent 24 h, the images of the cells were visualized at a 20× magnification using a Nikon microscope (Tokyo, Japan) fitted with a Leica digital camera. Untreated cells were set as a negative control.

### 3.9. Statistical Analysis

All results were subjected to statistical analyses. All data were expressed as means ± SD obtained from triplicate experiments. Statistical analysis was performed using the software of Statistical Analysis System Version 8.0 (SAS 8.0, Stat-Ease Inc., Minneapolis, MN, USA). Differences were considered statistically significant at *p* < 0.05.

## 4. Conclusions

In this study, an ultrasonic-assisted enzymatic extraction method was developed for the extraction of phenolic compounds from the stems of *T. quadrispinosa*, and the optimal extraction conditions were obtained by response surface methodology. The high correlation (*R*^2^ = 0.9926) of the model indicated that the second-order polynomial model could successfully express the influence of independent variables on the response. The optimal UAEE conditions (cellulase concentration of 1.74%, ultrasonic extraction time of 25.5 min and ultrasonic temperature of 49.0 °C) for the extraction process resulted the yield of 53.6 ± 2.2 mg·GAE/g·DW. The extracts obtained from UAEE presented highest antioxidant activities through ABTS, DPPH, TAC and FRAC methods, and were 1.54 ± 0.09 mmol Trolox equivalent (TE)/g·DW, 1.45 ± 0.07 mmol·TE/g·DW, 45.2 ± 2.2 mg·GAE/g·DW, and 50.4 ± 2.6 μmol FeSO_4_ equivalent/g·DW, respectively, and had the lowest IC_50_ values of 160.4 ± 11.6 μg/mL, 126.1 ± 10.8 μg/mL, and 178.3 ± 13.1 μg/mL against Hela, HepG-2 and U251 tumor cells, respectively, compared with other extraction methods including heat extraction, ultrasonic-assisted extraction and enzyme-assisted extraction. The results evidence that UAEE combined with RSM is an effective technique for extraction of phenolic compounds from the stems of *Trapa quadrispinosa* Roxb. The knowledge obtained from this study should be helpful for further exploitation and application of this resource.

## Figures and Tables

**Table 1:** Box-Behnken design with independent variables and its experimental response values.

Run	Independent Variables			*Y* (TPC mg·GAE/g·DW)	
	*X*_1_	*X*_2_	*X*_3_	Experimental	Predicted
1	−1	−1	0	50.3	50.60
2	−1	1	0	51.1	51.05
3	−1	0	−1	48.4	48.23
4	−1	0	1	47.6	47.53
5	1	−1	0	48.7	48.75
6	1	1	0	49.9	49.60
7	1	0	−1	47.6	47.68
8	1	0	1	44.6	44.78
9	0	−1	−1	46.6	46.48
10	0	−1	1	45.5	45.28
11	0	1	−1	47.5	47.73
12	0	1	1	45.2	45.33
13	0	0	0	52.7	52.33
14	0	0	0	52.4	52.33
15	0	0	0	51.9	52.33

**Table 2:** Analysis of variables for regression model of response in extraction conditions.

Source	Coefficient Estimate	Sum of Squares	*df*	Mean Square	*F* Value	*p* Value	Significance
Model	52.33	96.62	9	10.74	74.90	<0.0001	*** <0.001
*X*_1_	−0.82	5.44	1	5.44	37.99	0.0016	** <0.01
*X*_2_	0.33	0.84	1	0.84	5.90	0.0595	>0.05
*X*_3_	−0.90	6.48	1	6.48	45.21	0.0011	** <0.01
*X*_1_*X*_2_	0.10	0.040	1	0.040	0.28	0.6199	>0.05
*X*_1_*X*_3_	−0.55	1.21	1	1.21	8.44	0.0336	* <0.05
*X*_2_*X*_3_	−0.30	0.36	1	0.36	2.51	0.1739	>0.05
*X*_1_^2^	−0.74	2.03	1	2.03	14.17	0.0131	* <0.05
*X*_2_^2^	−1.59	9.35	1	9.35	65.26	0.0005	*** <0.001
*X*_3_^2^	−4.54	76.16	1	76.16	531.35	<0.0001	*** <0.001
Residual		0.72	5	0.14			
Lack of fit		0.39	3	0.13	0.80	0.5986	>0.05
Pure error		0.33	2	0.16			
Col Total		97.33	14				

Notes: * Significant at 0.05 level; ** Significant at 0.01 level; *** Significant at 0.001 level. *R*^2^ = 0.9926; *R*^2^*_Adj_* = 0.9794; C.V. = 0.78; Adeq Precision = 24.451.

**Table 3:** Comparison of experimental results obtained from different extraction methods.

Extraction Methods	Extraction Conditions				Response
	Cellulase Concentration (%)	Time (min)	Liquid to Solid Ratio (mL/g)	Temperature (°C)	TPC (mg/g)
HE	—	120	30	80	42.4 ± 1.3
UAE	—	30	30	50	48.2 ± 1.4
EAE	2.0	720	30	50	44.8 ± 1.5
UAEE	1.74	25.5	30	49	53.6 ± 2.2

**Table 4:** Independent variables and their levels used for the Box-Behnken design.

Independent Variables	Coded Levels		
	−1	0	1
Cellulase concentration (*X*_1_) (%)	1.5	2.0	2.5
Ultrasonic time (*X*_2_) (min)	20	25	30
Ultrasonic temperature (*X*_3_) (°C)	40	50	60
